# What Shapes the Phylogenetic Structure of Anuran Communities in a Seasonal Environment? The Influence of Determinism at Regional Scale to Stochasticity or Antagonistic Forces at Local Scale

**DOI:** 10.1371/journal.pone.0130075

**Published:** 2015-06-23

**Authors:** Clarissa de Araújo Martins, Fabio de Oliveira Roque, Bráulio A. Santos, Vanda Lúcia Ferreira, Christine Strüssmann, Walfrido Moraes Tomas

**Affiliations:** 1 Programa de Pós-Graduação em Ecologia e Conservação, Centro de Ciências Biológicas e da Saúde, Universidade Federal de Mato Grosso do Sul, Campo Grande, Mato Grosso do Sul, Brazil; 2 Centro de Ciências Biológicas e da Saúde, Universidade Federal de Mato Grosso do Sul, Campo Grande, Mato Grosso do Sul, Brazil; 3 Departamento de Sistemática e Ecologia, Universidade Federal da Paraíba, João Pessoa, Paraíba, Brazil; 4 Departamento de Ciências Básicas e Produção Animal, Faculdade de Agronomia, Medicina Veterinária e Zootecnia, Universidade Federal de Mato Grosso, Cuiabá, Mato Grosso, Brazil; 5 Laboratório de Vida Selvagem, Embrapa Pantanal, Empresa Brasileira de pesquisa Agropecuária, Corumbá, Mato Grosso do Sul, Brazil; National & Kapodistrian University of Athens, Faculty of Biology, GREECE

## Abstract

Ecological communities are structured by both deterministic and stochastic processes. We investigated phylogenetic patterns at regional and local scales to understand the influences of seasonal processes in shaping the structure of anuran communities in the southern Pantanal wetland, Brazil. We assessed the phylogenetic structure at different scales, using the Net Relatedness Index (NRI), the Nearest Taxon Index (NTI), and phylobetadiversity indexes, as well as a permutation test, to evaluate the effect of seasonality. The anuran community was represented by a non-random set of species with a high degree of phylogenetic relatedness at the regional scale. However, at the local scale the phylogenetic structure of the community was weakly related with the seasonality of the system, indicating that oriented stochastic processes (e.g. colonization, extinction and ecological drift) and/or antagonist forces drive the structure of such communities in the southern Pantanal.

## Introduction

Which forces shape biodiversity patterns? This is one of the central questions driving many biodiversity studies [1]. For many years, community ecology studies focused largely on trying to understand the role that deterministic processes, based on the niche concept [2], played in the assembly of biological communities. In recent decades, this view has been modified mainly by the recognition that communities are organized at multiple scales, and that oriented stochastic processes, such as chance colonization, random extinction and ecological drift also play important roles in the assemblage of communities [3–6]. Moreover, ecologists are increasingly adopting an evolutionary perspective, with the phylogenetic community approach providing insight into how evolutionary processes may have shaped contemporary patterns of biodiversity, untangling the role of historical contingency, niche-based processes and neutral processes [7].

Recent evidence suggests that stochastic and deterministic processes tend to act simultaneously on the organization of biological communities [7,8], and that they can have different relative roles across scales [9–11]. Moreover, when stochastic processes such as events related to death, birth and dispersion are the main drivers of community assemblage [3], the species tend to co-occur regardless of their phylogenetic relatedness. This results in a community structure that is phylogenetically random [12,13]. Deterministic processes may be categorized into two assembly rules involving environmental filtering and density-dependent biotic interactions [7]. When environmental conditions are the dominant ecological forces structuring communities, unsuitable conditions filter out species that do not have morphological, physiological, and/or ecological adaptations to persist and reproduce under such conditions [14]. Only species possessing such attributes will persist in the community. Because niche is generally conserved along a phylogeny [15], this phenotypic attraction driven by environmental conditions results in co-occurrence of close relatives and phylogenetic clustering at the community level [14,16,17]. The second assembly rule is widely known as limiting similarity, via the assumption of niche conservatism, works on the principle that phylogeny should be a good proxy for similarity in ecological niche of species [7,18]. This principle, inherited from Charles Darwin’s observations, assumes that competition is higher among close relatives, limiting their co-occurrence and leading to the competitive exclusion of extremely similar species [19]. In this case, competitive exclusion limits the co-occurrence of phylogenetically close species [18,20]. The permanence of species within a community is thereby determined principally by their ability to interact (negatively or positively) with other members of the community, instead of being related to their capability to survive and reproduce under unsuitable conditions [7]. Communities organized by such density-dependent processes tend to have distant relatives and to be phylogenetically overdispersed [21,22].

Observational studies have been diverse in their approaches towards phylogenetic structure of communities, but most have focused on the terrestrial systems, particularly with plant communities [23]. To our knowledge, the role of seasonally fluctuating environments on the phylogenetic structure of animal communities remains untested. Seasonally flooded environments are suitable to address temporal aspects in the study of community phylogenetics, since alternation of dry and flood periods promote huge environmental variations [24]. One example of such a seasonal environment, where recurrent droughts and floods play a key ecological role, is the Pantanal floodplain in South America. This region is considered one of the largest continuous wetlands on the globe, with energy, nutrient, and hydrologic dynamics influenced by flood pulses, for which every single species has its own array of survival strategies [25]. In systems with periodic environmental fluctuations, phylogenetic structure of biological communities may experience temporal changes, becoming more clustered and overdispersed in periods of unsuitable and suitable conditions, respectively. Environmental fluctuations could act as a filter that select key traits in community members and, if they are correlated with species relatedness, close related species should have similar sensitivities to disturbance [26].

In this study, we examined the phylogenetic structure of anuran communities in the Pantanal wetland in two complementary ways under the premise that patterns and processes are scale-dependent [27]. Firstly, we assessed whether Nhecolândia—one of the sub-regions of the Pantanal subject to shorter and less extensive floods, with a sub-humid and marked seasonal climate [28]–hosts a non-random subset of anuran species. We expect that the phylogenetic structure of communities of anurans from Nhecolândia would be more clustered than expected by chance.

Secondly, we evaluated how the anuran phylogenetic arrangement at Nhecolândia varies according to the fluctuating conditions (dry-flood periods) in the sub-region. Our objective was to identify the role of either deterministic (environmental filtering or ecological interactions) or oriented stochastic processes in the assembly of anuran communities. While precipitation may be the main extrinsic factor controlling the activity and breeding of anurans, and consequently, the organization of anuran communities in tropical regions [29–32], we expected that during the drier periods the environmental filtering would be the main structuring force, resulting in the co-occurrence of close relatives and in a clustered phylogenetic pattern. In addition, in line with this framework, we expected high phylogenetic beta diversity between seasonal periods with taxa turnover being derived mostly by close relatives.

## Methods

### Study area

**Fig 1:**
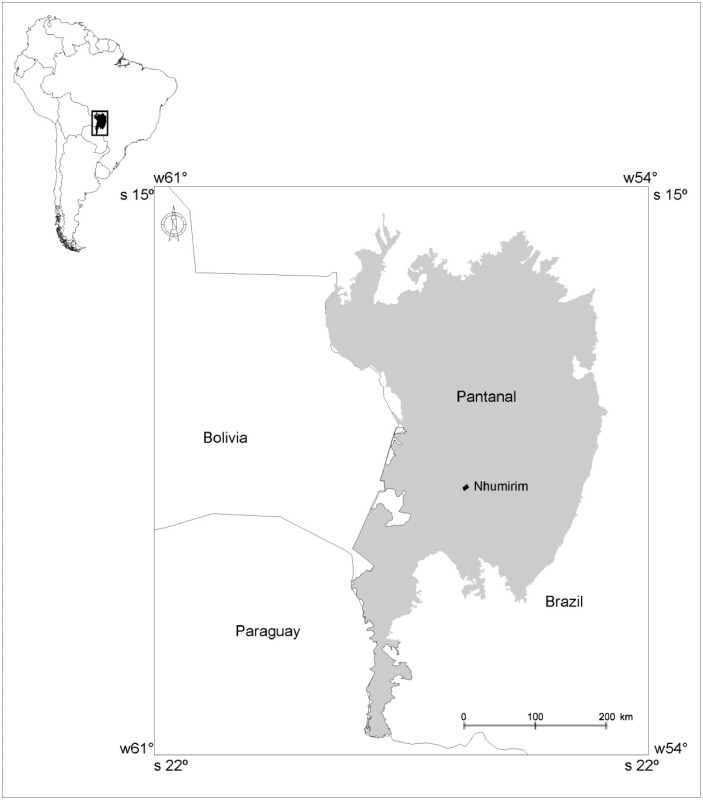
Location Nhumirim Ranch. Location in South America of the study area, Nhumirim Ranch (square), in Pantanal floodplain of Brazil.

The Pantanal is one of the biggest wetlands in the world and has a marked the dry/flood seasonal regime. Differences in the magnitude and frequency of floods, soil, and vegetation, among others, confer unique characteristics to each of several sub-regions recognized in the Brazilian Pantanal [33]. Field study was conducted at Nhumirim Ranch experimental station—EMBRAPA—Pantanal (18°59’ S; 56°39’ W; municipality of Corumbá, state of Mato Grosso do Sul, Brazil), in the Nhecolândia sub-region [34]. This (hereafter called Nhecolândia; Fig 1) is one of the driest sub-regions of the Pantanal. The area is characterized by a mosaic of both brackish and freshwater ponds, interspersed with forested patches growing on non-floodable terrains. Situated at an interfluve, the Nhumirim Ranch—and in fact, almost the entire Nhecolândia sub-region—has a low annual precipitation and is under limited influence riverine flood pulses [28].

### Data collection

Collection procedures, including capture, restraint, marking and release are in accordance with Resolution 301/2012 of the Regional Council of Biology—CRBio, which concerns the ethical protection of local wildlife. Consequently, specimens were collected and preserved only when we required confirmation of species taxonomic resolution. Among the species that were preserved none were considered endangered and/or rare. During the time of our collection of specimens, there were no requirements from the Brazilian government for previous submissions ethics committee concerning methods for specimen euthanasia. The only requirement was, at the time, a license for capture, collection and transportation of specimens. This requirement was fully met.

Voucher specimens were collected, anesthetized with Thiopental and killed in 10% alcohol (according to the Resolution of the Federal Council of Biology—CFBio No. 301, December 8, 2012), and fixed in 10% formalin. They were subsequently stored in 70% alcohol and deposited in the Zoological Collection of the Universidade Federal de Mato Grosso do Sul (ZUFMS, at Campo Grande, Mato Grosso do Sul) and in the Zoological Collection of Vertebrates of the Universidade Federal de Mato Grosso (UFMT, at Cuiabá, Mato Grosso). All procedures relating to collection procedures, including capture, restraint, marking, release and specimen preparation were made under IBAMA license 029/04, Process: 02010.002379/ 04–31 and SISBIO/ICMBio n° 10640.

The dataset that used was extracted from the ‘Herpetofaunal Monitoring Project’ conducted at Nhumirim Ranch from 2005 to 2006 within the PROBio program sponsored by the Ministerio do Meio Ambiente (MMA), and from 2007 to 2013 supported by a Embrapa Pantanal partnership project. This dataset includes information on abundances of amphibians, together with environmental variables. Sampling and measured environmental variables are detailed elsewhere [35].

Sampling was conducted over 24 field expeditions between February 2005 and December 2012. Pitfall traps with drift-*fences* were used to capture anurans. Plastic buckets (100 litres each) in a pitfall set were arranged in “Y” in the first nine expeditions (along 24 sampling points), and in line, in subsequent expeditions (in additional 23 sampling points). Pitfall trap sets in Y consisted of four buckets, while those in line consisted of two buckets, placed approx. 10 m apart and interconnected by a plastic drift fence 1 m high, with a one meter overlap at each end [36].

**Fig 2:**
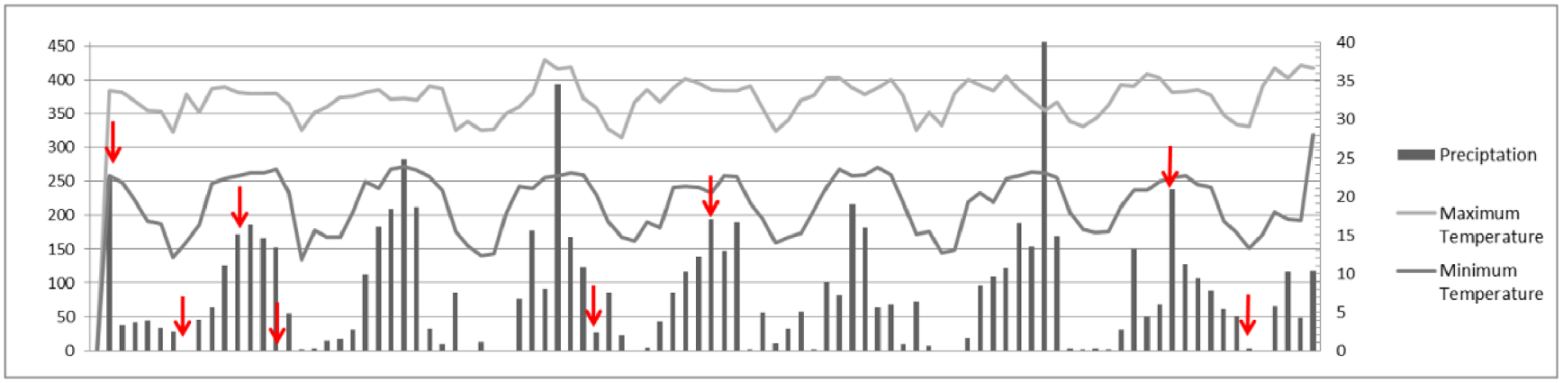
Meteorological data from the Nhumirim Ranch. Precipitation (left axis) and maximum and minimum average temperatures between February 2005 and December 2012 (right axis). The arrows indicate the peak periods of drought and rain that we selected for the analysis.

We selected data from eight field expedition collections that represented peaks of rainy and dry periods (rainy: 11/Feb/05, 10/Jan/06, 22/Nov/08, 17/Jan/12; dry: 09/Ago/05, 17/May/06, 16/Apr/08, 24/Sep/09). In order to evaluate the highest and lowest precipitation periods, we used data on historical precipitation, and maximum and minimum temperatures from the Instituto Nacional de Metereologia, INMET (National Institute of Meteorology) website, from its data base BDMEP (Bank of Meteorological Data for Education and Research), specifically from the meteorological station located at Nhumirim Ranch (http://www.inmet.gov.br/portal/) (Fig 2).

### Regional phylogeny

In order to compare the phylogenetic structure of the anuran community at Nhecolândia with those from the Pantanal as a whole, we used a list of all anurans already identified in the southern part of the Pantanal wetland [37] and selected those that could occur at Nhecolândia and were likely to be collected by our sampling method (see Letcher [12] for a similar procedure with plant species). Our resulting regional pool contained 44 species of potential colonizer anurans from the Pantanal region.

To assess the phylogenetic similarities among all species in our dataset, we used a cladogram based on the time-calibrated tree proposed by Pyron & Wiens [38], which contains 2,871 anuran species, we received the newick file from the authors. We pruned the time-calibrated tree to include only the anuran species found in our study. First, we inputted the newick file of the phylogeny proposed by Pyron & Wiens [38] into Phylocom software. Using the function Phylomatic [38,39], we pruned the input tree to match the set of taxa included in our community. The species from the regional pool that were not included in the phylogenetic hypothesis of Pyron & Wiens [38] were replaced by sister species [40–42]. In total we replaced four species of the local pool and six species of the regional pool. This procedure is valid since sister species within a phylogeny have similar phylogenetic distance. In order to evaluate the phylogenetic structure of anuran communities from Nhecolândia on a local scale, we built the regional species pool based on the species recorded during field expeditions in both dry and rainy seasons.

### Community phylogenetic structure and statistical analyses

We used Phylocom 4.2 software [43] to assess the phylogenetic structure of anuran communities from the Pantanal at different scales. Based on presence/absence and abundance metrics, we used two standardized indices proposed by Webb et al. [14]: the Net Relatedness Index (NRI), and the Nearest Taxon Index (NTI). These indices are calculated from the following formulas:

$$NRI_{obs}=-\frac{\left(MPD_{obs}-MPD_{null}\right)}{sdMPD_{null}}$$

$$NTI_{obs}=-\frac{\left(MNTD_{obs}-MNTD_{null}\right)}{sdMNTD_{null}}$$

where MPD_obs_ is the mean phylogenetic distance observed in the community, based on the length of the phylogeny branches, and MNTD_obs_ is the phylogenetic distance to the nearest taxon in the phylogeny. The values MPD_null_ and MNTD_null_ are obtained through sample randomizations generated by the null model. We used the null model "0" (Phylogeny Shuffle) for 999 randomizations. This model shuffles species labels across the entire phylogeny and randomizes phylogenetic relationships among species [43]. It is more appropriate for temporal analyses than other null models (e.g. Phylocom model 2) because it maintains sample abundance distribution, species richness, occupancy rates, conserves the spatial contagion of species and therefore also maintains the identity of surviving individuals between consecutive expeditions [44,45].

Assuming that the species traits are preserved along the phylogeny (phylogenetically conserved ecological traits sensu [7,46], significant positive values of NRI and NTI indicate that the species are phylogenetically closer than expected by chance (phylogenetic clustering) [47]. Conversely, significant negative values indicate that species are phylogenetically less related than expected by chance (phylogenetic dispersion). Non-significant values may indicate a random pattern [12,14]. While the NRI shows the phylogenetic distance within the basal branches of the phylogeny, the NTI shows the terminal structure of the phylogeny [47,48]. To test the significance level of NRI and NTI from a null model, we divided the number of null communities (MPD_null_) with MPD values that were higher or lower than the observed value (MPD_obs_) by the number of randomizations +1. If the generated value in the observed value was below 5% of the distribution generated by the null model, the NRI or NTI were considered significant [12,44].

We used a permutation test to assess whether the mean difference in values of NRI and NTI in the dry and rainy months was given by chance. First, we calculated the observed mean difference in values of NRI and NTI in the dry and rainy months. Then, we randomized 1000 times the NRI and NTI values between seasons and re-calculated the mean differences creating a random distribution to compare our observed value in relation to this distribution. We inferred the likelihood of the mean differences given by chance, taking α = 5%.

We compared the different periods in relation to phylobetadiversity patterns (phylogenetic distance among communities) using mean values of standardized phylobetadiversity based on presence/absence and abundance metrics (betaNRI_p/a_, betaNRI_ab_, betaNTI_p/a_, betaNTI_ab_). Negative values of betaNRI and betaNTI indicate high phylogenetic turnover between pairs of communities, and positive values indicate low turnover [49,50]. Phylobetadiversity tests were calculated using the COMDIST and COMDISTNT functions in Phylocom see. 4.1 [43]. One-way ANOSIM was used to test for differences in the phylobetadiversity between sampled periods.

## Results

**Fig 3:**
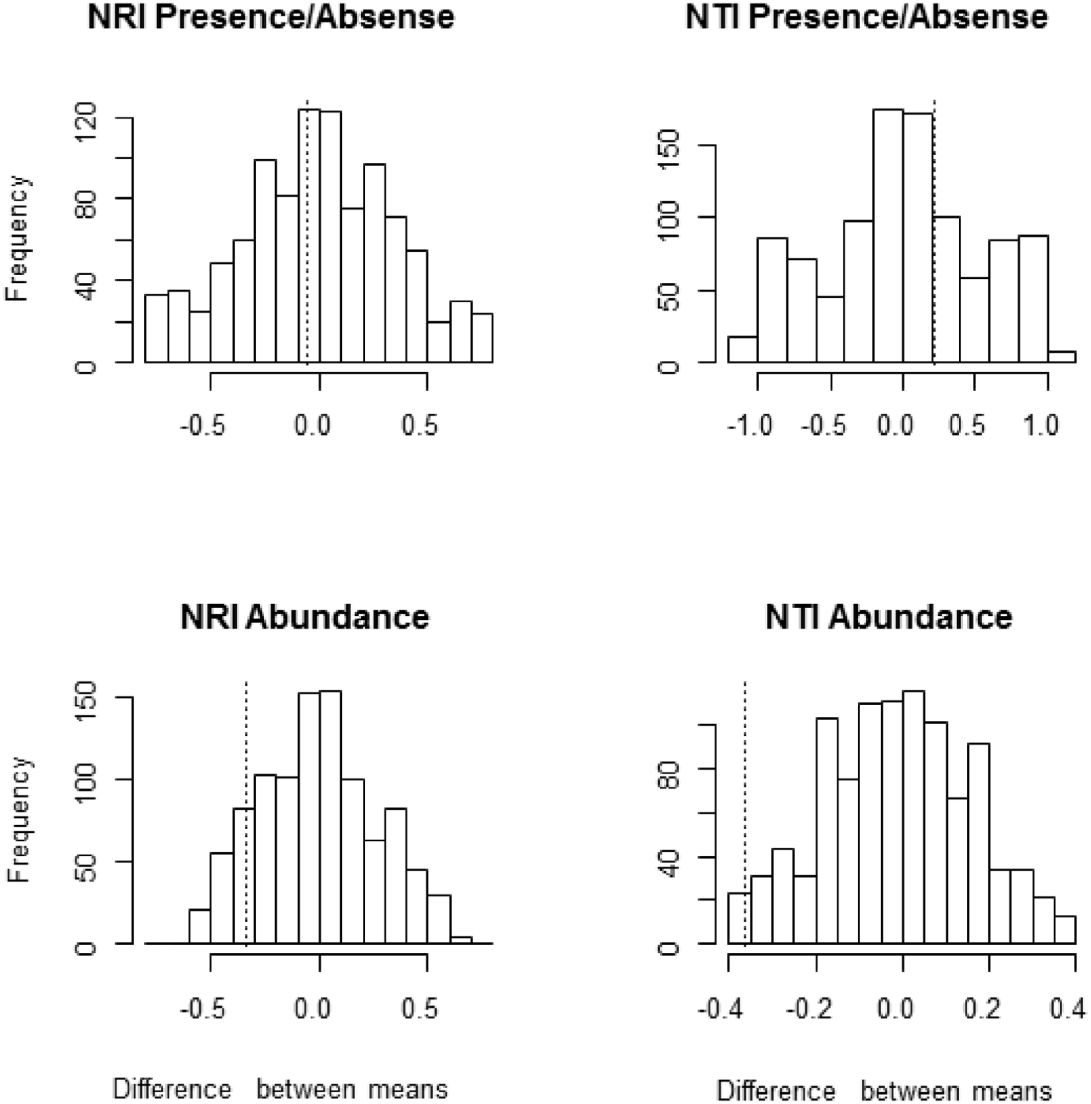
NRI and NTI values between seasons. Permutation test using mean difference in indices of phylogenetic structure of anuran communities, NRI and NTI, in rainy and dry seasons in the Nhecolândia region, Pantanal wetland, Brazil. The dashed lines indicate the actual mean difference between the periods (nri p/a p = 0.102, nti p/a p = 0.384, nri abd p = 0.076, nti abd p = 0.006).

The 9541 anurans recorded at Nhecolândia came mostly from the Leptodactylidae and Microhylidae, families which account for about a third of the recognized anuran species diversity in the southern Brazilian Pantanal (44 species). Considering the mean phylogenetic distance to the nearest taxon, the 16 species comprising the Nhecolândia anuran community were significantly more closely related than randomly expected from the 44 species pool of potential colonizers (NTI = 1.73, p = 0.004), indicating that Nhecolândia harbors a non-random, phylogenetically clustered subset of the Pantanal anurans. This trend disappeared when we considered the overall measure of phylogenetic distance (NRI = -0.14; p>0.05), suggesting that basal clades for Nhecolândia anura are random samples from the overall Pantanal pool. The permutation test showed significant difference of mean values only for NTI (p = 0.006) using abundance data (Fig 3).

Temporal shifts in phylogenetic community structure for Nhecolândia anura varied greatly and occurred irrespective of seasonality (Table 1). Although species richness was on average greater in the rainy than in the dry season, the phylogenetic structure of most communities did not differ significantly from random samples drawn from the 16 species pool of Nhecolândia anurans. Nonetheless, all but one of the NTI values were positive, indicating that a general trend of phylogenetic clustering was maintained over time (Table 1). NRI varied more across seasons, though a trend of clustering similar to that of NTI appeared when species abundance was taken into account.

**Table 1:** NRI and NTI index values for the anuran communities in rainy and dry periods at Nhumirim Ranch, Pantanal wetland, Brazil, using presence/absence and abundance metrics.

Presence/absence			Abundance		
Period	NRI	NTI	Period	NRI	NTI
Rainy	0.1359	1.9516*	Rainy	-0.9817	0.5106
Dry	-0.0595	0.1599	Dry	0.389	0.717
Rainy	-1.1066	0.6295	Rainy	0.1008	0.4739
Dry	-0.8234	0.724	Dry	0.591	1.1655*
Rainy	0.3019	0.281	Rainy	0.5092	0.6763
Dry	0.1466	1.6275*	Dry	0.114	1.0175
Rainy	-0.5016	0.3209	Rainy	0.7891	0.6765
Dry	-0.1898	-0.2023	Dry	0.6942	0.883

*Significant values.

The phylobetadiversity results indicated low turnover of pairs of communities (betaNRI and betaNTI positive values), except for betaNRI_p/a_ which showed high turnover of peer communities (negative values). ANOSIM analysis showed no significant differences in phylobetadiversity patterns for betaNRI_ab_ (R -0.3125, p = 1), betaNRI_p/a_ (R -0.010, p = 0652), betaNTI_ab_ (R 0.041,p = 0.304), and betaNTI_p/a_ (R -0.010, p = 0.552) (Table 2).

**Table 2:** Values of standardized phylobetadiversity (betaNRI and betaNTI) in Dry-Dry, Rainy-Rainy, and Dry-Rainy periods.

Variable	Periods	Mean
NRI presence-absence	Dry-Dry	-0.752*
NRI presence-absence	Rainy-Rainy	-1.1547*
NRI presence-absence	Dry-Rainy	-0.9632*
NRI abundance	Dry-Dry	0.6895*
NRI abundance	Rainy-Rainy	-0.5004
NRI abundance	Dry-Rainy	0.3674
NTI presence-absence	Dry-Dry	1.9072*
NTI presence-absence	Rainy-Rainy	1.1273*
NTI presence-absence	Dry-Rainy	1.3810*
NTI Abundance	Dry-Dry	1.9631*
NTI Abundance	Rainy-Rainy	1.5786*
NTI abundance	Dry-Rainy	1.9024*

Symbols indicate statistical significance of a t-test comparing observed values versus expected value of zero for random data (*p<0.05).

## Discussion

Phylogenetic clustering has been described a dominant pattern in studies of phylogenetic structure of communities [51], underpinning the idea that environmental conditions guide the assembly of communities [16,22,47]. In this study, we found two main results: 1) the anuran community from the Nhecolândia study site is a non-random sample of the anuran species pool from the southern Pantanal, and comprises 16 species more phylogenetically related than expected by chance; 2) On a local scale, the temporal shifts in phylogenetic structure cannot be predicted by seasonality, as stochastic processes and/or antagonistic forces seem to guide the assemblage of anuran communities in the region.

Although phylogenetic clustering may result from competition among distant relatives [52,53], hierarchical competition [54], facilitation/mutualism among close relatives [55] and other density-dependent processes that operate most intensively at the neighborhood scale [7], the bulk of evidence still indicates that environmental filtering determines phylogenetic clustering at broader spatial scales [56,57]. In fact, recent studies have shown strong signals of evolutionary processes in contemporary amphibian distributional patterns [40,58,59,60]. In a spatial context, historical events, current climatic conditions and geographical distances are complementary predictors of amphibian composition even for sites within the same biome, such as Atlantic Forest [60]. In the case of the Pantanal, when comparing the abiotic conditions of the Nhecolândia sub-region with the region overall, we clearly found substantial differences in climatic conditions for anurans. Nhecolândia is one of the most arid sub-regions in the Pantanal and less influenced by the flooding pulse than elsewhere in the biome. In the rainy season only 30% of the area is flooded [28], resulting in long dry periods and floods that are unpredictable when compared to more humid and stable regions elsewhere in the Pantanal. This further supports the idea that phylogenetic variation in amphibian assemblages can be related to climatic regional stability [60].

The analysed Nhecolândia species pool is dominated by species from the families Microhylidae and Leptodactylidae. In the Pantanal, most anuran species have a more generalist reproductive mode (mode 1 sensu [61]), which involves high reproductive investment (large clutches), fast larval development [62], and breeding during the rainy season. Such species have an explosive breeding pattern (with rapid response to periods of rain), and most of them lay eggs in temporary ponds, embedded in foam nests (Leptodactylidae) to prevent desiccation when such ponds dry out [62]. These features can also be viewed as an evolutionary response to exploit unpredictable and seasonal environments, including temporary ponds in Brazilian savanna and Chaco [62–64]. This set of attributes gives these groups higher breeding success in seasonal and unpredictable environments such as those studied by us in the Nhecolândia sub-region of the Pantanal wetland. Low values phylogenetic community turnover between sites within unstable regions seems to be related to homogenization of taxonomic and phylogenetic composition within a region that harbors amphibian species with broad range sizes [60]. In our case, the low turnover detected when considering the abundance of the taxa can also be related to the broad range sizes of most anuran species inhabiting the Pantanal (see Junk et al. 2006 [25]). However, it can also be related to the recurrent increase in connectivity amongst aquatic habitats in floodplains and to the consequent habitat homogenization imposed by the floods (e.g., Thomaz et al. 2007 [65]). Such processes can modify local distribution patterns of anurans and diminish the influences of species specific attributes and of other environmental factors.

On a local scale, however, there is unclear phylogenetic clustering and dispersion pattern in the anuran communities between flood and dry seasons, as indicated by the large variation between the deployed metrics. We obtained random samples of communities of anurans when using presence and absence data, suggesting that the temporal variation in the phylogenetic structure of the communities is unclear. In this case, there may be a lack of deterministic rules guiding the assemblage of the studied communities and, therefore, oriented stochastic processes may be the primary structuring force [3,14,66]. Alternatively, it is also possible that antagonistic deterministic forces contribute similarly to the assembly of anuran communities. Lovette & Hochatchka (2006) [67] suggest that whenever a random pattern is detected in a community phylogenetic structure, it is likely that opposing forces such as ecological interactions and abiotic filters are annulling each other, generating a random pattern [68]. Considering that anurans strongly interact for resources or shelter [69,70], and that the local environmental conditions (e.g. presence of water) simultaneously set limits to species co-occurrence, both deterministic forces would be expected to act in the assemblage of these communities. Nevertheless, when we used abundance data for the NTI permutation test, the resultant value differs from random, and there is an apparent phylogenetic clustering of the means in all seasons, though higher during dry periods. Under such circumstances, the ecological processes driving phylogenetic clustering are operating predominantly on the variation of dominance of phylogenetically close species. Indeed, the phylobetadiversity indexes sensitive to phylogenetic gradients related more to terminal nodes (COMDISTNT), also showed that variation between the samples are caused by low turnover of the most abundant terminal taxa (positive value of NTI). However, as we found no differences between all measures of phylobetadiversity within and between seasons, environmental seasonality alone (as a proxy for environmental filter) is not enough to account for the phylogenetic patters of most abundant close relatives at the local scale. Consequently, other underlying process (e.g. chance colonization, hierarchical ecological interactions) could play a role in the assemblage of the anuran communities in this region of Nhecolândia.

## Conclusion

We conclude that the role environmental conditions determining community membership in a seasonal environment is scale dependent. On a regional scale, the abiotic conditions at the Nhecolândia site seem to filter out a set of anuran species known to occur in the southern Pantanal wetland, allowing the coexistence of a small group of close relatives. On a local scale however, the stochastic processes and/or antagonistic forces seem to guide the assemblage of anuran communities, since their phylogenetic structure is highly variable and poorly predictable in the dry and rainy seasons.

## Supporting Information

S1 Fig Regional Pool of Species of the Pantanal. Dendrogram generated from the regional pool of species of the Pantanal wetland, Brazil (A), and species occurring in the Nhecolândia region of the Pantanal (B) (Mesquite 2.01).
(TIF)

S1 Table Dataset of anura. Showing the database used for carrying out the phylogenetic structure of the communities of anurans from Nhecolândia sub-region (Pantanal wetland, state of Mato Grosso do Sul, Brazil). * Species were replaced by species-sisters for building the regional phylogeny [30].
(XLSX)
