# Correction: What Shapes the Phylogenetic Structure of Anuran Communities in a Seasonal Environment? The Influence of Determinism at Regional Scale to Stochasticity or Antagonistic Forces at Local Scale

**DOI:** 10.1371/journal.pone.0151734

**Published:** 2016-03-24

**Authors:** Clarissa Araújo Martins, Fabio Oliveira Roque, Bráulio A. Santos, Vanda Lúcia Ferreira, Christine Strüssmann, Walfrido Moraes Tomas

The first and second authors’ names appear incorrectly in the citation. The correct citation is: Martins CA, Roque FO, Santos BA, Ferreira VL, Strüssmann C, Tomas WM (2015) What Shapes the Phylogenetic Structure of Anuran Communities in a Seasonal Environment? The Influence of Determinism at Regional Scale to Stochasticity or Antagonistic Forces at Local Scale. PLoS ONE 10(6): e0130075. doi: 10.1371/journal.pone.0130075.
