# Supplementary figures and images for: What Shapes the Phylogenetic Structure of Anuran Communities in a Seasonal Environment? The Influence of Determinism at Regional Scale to Stochasticity or Antagonistic Forces at Local Scale
Source: PLoS One. 2015 Jun 23;10(6):e0130075. doi: 10.1371/journal.pone.0130075 (PMC4478043; doi:10.1371/journal.pone.0130075)

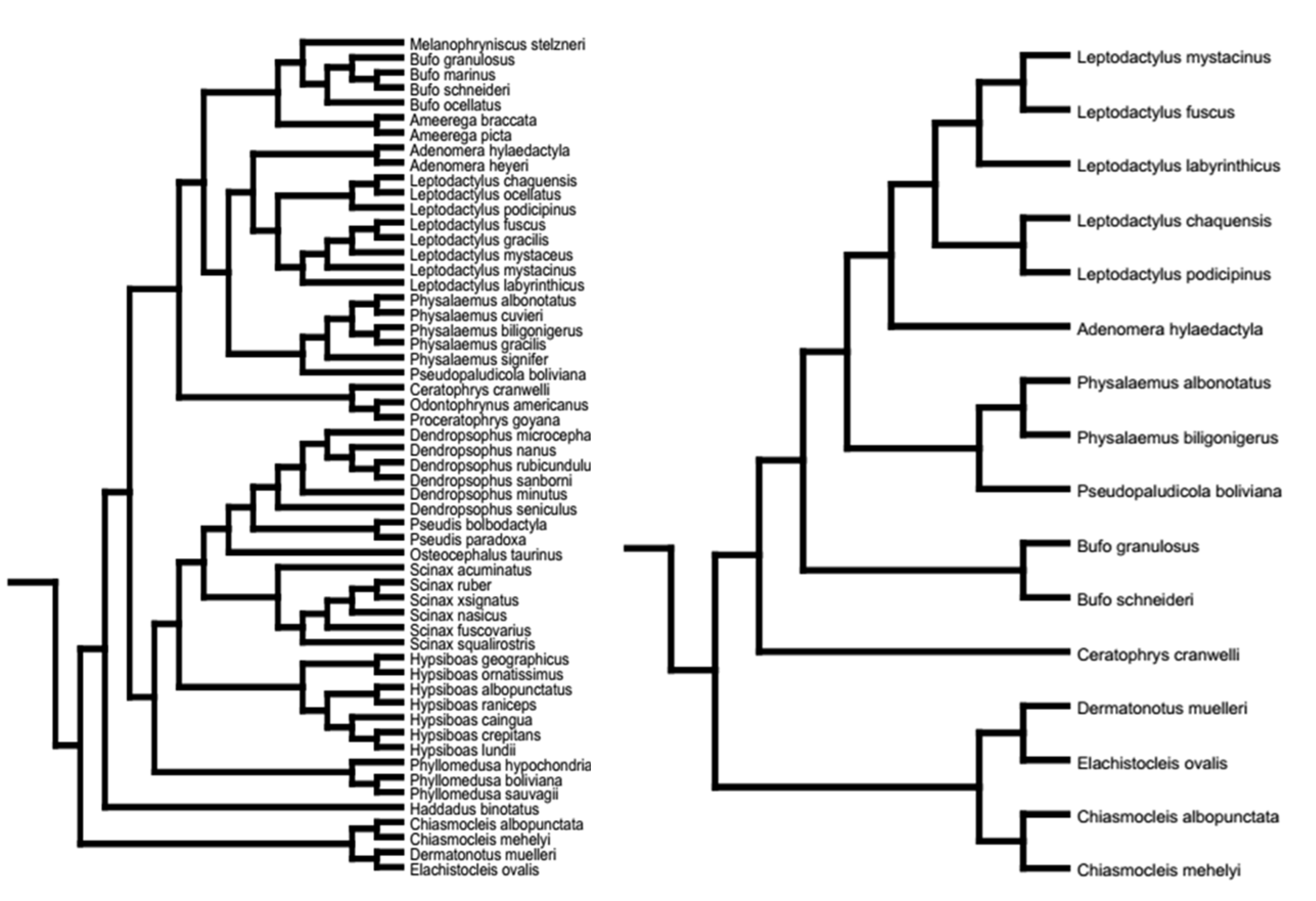

Supplement: S1 Fig — Dendrogram generated from the regional pool of species of the Pantanal wetland, Brazil (A), and species occurring in the Nhecolândia region of the Pantanal (B) (Mesquite 2.01). (TIF) [file pone.0130075.s001.tif]
